# The potential role of m6A reader YTHDF1 as diagnostic biomarker and the signaling pathways in tumorigenesis and metastasis in pan-cancer

**DOI:** 10.1038/s41420-023-01321-4

**Published:** 2023-01-28

**Authors:** Yanan Zhu, Jing Li, Hang Yang, Xinyi Yang, Ya Zhang, Xinchao Yu, Ying Li, Gangxian Chen, Zuozhang Yang

**Affiliations:** 1 Bone and Soft Tissue Tumors Research Centre of Yunnan Province, Department of Orthopaedics, The Third Affiliated Hospital of Kunming Medical University (Yunnan Cancer Hospital), 650118 Kunming, Yunnan China; 2 Department of Rehabilitation Medicine, The Second Affiliated Hospital of Kunming Medical University, 650106 Kunming, Yunnan China; 3 Guizhou Medical University, 550004 Guiyang, Guizhou China

**Keywords:** Oncogenes, Metastasis

## Abstract

m6A is an important RNA methylation in progression of various human cancers. As the m6A reader protein, YTHDF1 is reported to accelerate m6A-modified mRNAs translation in cytoplasm. It is highly expressed in various human cancers and contributes to the progression and metastasis of cancers. YTHDF1 was closely associated with poor prognosis and also used as a molecular marker for clinical diagnosis or therapy in human cancers. It has been reported to promote chemoresistance to Adriamycin, Cisplatin and Olaparib by increasing mRNA stability of its target molecule. Moreover, it contributes to CSC-like characteristic of tumor cells and inducing the antitumor immune microenvironment. Here, we reviewed the clinical diagnostic and prognostic values of YTHDF1, as well as the molecular mechanisms of YTHDF1 in progression and metastasis of human cancers.

## Facts

- YTHDF1 is overexpressed in various human cancers.
- YTHDF1 is used as a molecular marker for clinical diagnosis or prgnosis in human cancers.
- YTHDF1 exerted an important role in tumorigenesis and metastasis of cancers by different mechanisms.

## Open questions

- Whether YTHDF1 is used as the diagnostic or prognostic biomarker of cancers?.
- How YTHDF1 regulates the progression and metastasis of human cancers?
- What’s the targeted genes that regulated by YTHDF1 in tumorigenesis and metastasis of human cancers.

## Introduction

RNA modification is a key process of post-transcriptional regulation of genes in epigenetics [1]. m6A is a commonly seen post-transcriptional RNA modification and first discovered in 1974, referring to the occurrence of adenine in RNA. The m6A methylation modification accounts for more than 80% of all RNA methylation [2, 3]. m6A modification can regulate various physiological processes such as cell division, maintenance and differentiation of stem cell characteristics, immune homeostasis, mitosis, gametogenesis, sex determination, and biological rhythms by regulating RNA metabolism [4]. In recent years, studies have found that m6A-regulated genes are closely related to various diseases, including various cancers, neurological, cardiovascular diseases, and infections and other immune system diseases [5–9]. It is mainly enriched in the 3’ UTRs close to the stop codon of mRNA, which occurs at specific RRACH consensus sequences (R: A/G, H: A/C/U). It has been found that several non-coding RNAs such as lncRNA, rRNA and spliceosome RNA also have a large number of m6A modification activities before and after transcription [10].

**Fig. 1:**
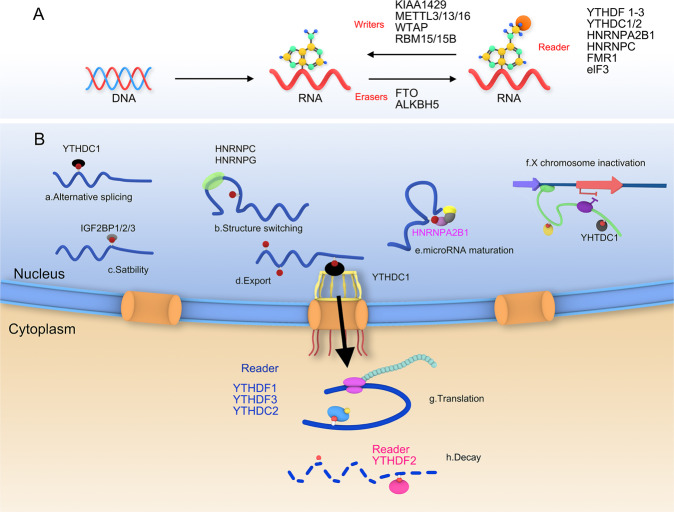
m6A methylation modification. **A** The methylation modification process of m6A is reversible and requires the participation of methyltransferases, demethylases and methylation reading proteins. Methylase including METTL3/13, WTAP, RBM15 etc. The main role is to catalyze the m6A modification of adenylate on mRNA. Demethylases, including FTO and ALKHB5, are used to demethylate bases that have undergone m6A modification. The main function of reader proteins including YTHDF1/2/3, YTHDC1/2 etc. is to recognize bases with m6A modification, thereby activating downstream regulatory pathways, such as RNA degradation, miRNA processing, etc. **B** The molecular biological function regulated by m6A methylation modification, including alternative splicing, structure switching, stability, export, microRNA maturation, X chromosome inactivation, translation and mRNA decay etc.

m6A methylation modification is a reversible process and basically the same as the methylation modification of DNA and histones, and the overall methylation level in RNA is adjusted by transmethylation and demethylation [11–13]. The m6A methylation-regulated proteins includes three categories, namely writers, erasers, and readers. Writers are also known as methyltransferases, which include Wilm’s tumor 1-associated protein (WTAP), RNA Binding Motif Protein 15(RBM15), Methyltransferase 3(METTL3), METTL14, METTL16, RBM15B, zinc-finger CCCH domain-containing protein 13(ZC3H13), etc., are mainly responsible for the methylation process of RNA, and catalyze the occurrence of the sixth nitrogen atom of adenosine on mRNA [14]. Erasers corresponding to Writers are also called demethylases, mainly including AlkB homolog 5(ALKBH5) and alpha-ketoglutarate-dependent dioxygenase (FTO). They can reverse the methylation process, thereby reducing methylation in RNA level of ization. Readers (m6A-binding proteins) are proteins that recognize methylation, also known as methylated reading proteins, such as IGF2BP1/2/3, YTHDF1/2/3, YTHDC1/2, heterogeneous nuclear ribonucleoprotein A2B1(HNRNPA2B1) [6, 15–19]. m6A-binding protein usually contains YT521-B homology (YTH) domain, YTH protein is the most deeply studied m6A-binding protein, mainly including YTHDF1, YTHDF2 in the cytoplasm, YTHDF3 and YTHDC1-2 in the nucleus [20–24]. As shown in Table 1, the m6A modification controls the fate of the modified RNA by interacting with different recognition proteins “readers”, such as activating mRNA translation or degradation, and accelerating mRNA export from the nucleus [25, 26] (Fig. 1).

**Table 1:** The location and function of m6A readers.

Readers	Location	Function
YTHDC1	Nucleus	Alternative splicing, nuclear export, X chromosome inactivation
YTHDC2	Cytoplasm	Translation, decay
HNRNPC	Nucleus	Structure switching, pre-mRNA processing
HNRNPG	Nucleus	Structure switching
IGF2BP1/2/3	Nucleus	Stability
HNRNPA2B1	Nucleus	miRNA maturation
YTHDF1	Cytoplasm	Translation
YTHDF2	Cytoplasm	Decay
YTHDF3	Cytoplasm	Translation, decay,

## YTH domain family-m6A reader

Except for the YTH domain, the members of the YTH domain family of proteins have no amino acid sequence similarity, and there are also significant differences in protein size and domain arrangement [27, 28]. For example, although YTHDC1 and YTHDC2 have similar names, they are not homologous except that they both have a YTH domain [29]. It is generally believed that the YTHDC1-2 protein is mainly involved in the alternative splicing regulation of pre-mRNA in the nucleus, and the related “readers” that regulate protein abundance in the cytoplasm by post-transcriptional regulation mainly include YTHDF1, YTHDF2, and YTHDF3 [30]. Currently known studies have shown that the “reader” YTHDF1 can recruit eukaryotic initiation factor eIF3 family members by binding to m6A modification site in 3’- UTR region of mRNA, and directly initiate G-cap-independent translation [31]. In addition, YTHDF2 promotes mRNA deadenylation by recognizing m6A modification sites and recruiting CNOT1-CCR4 complexes, thereby reducing the stability of mRNA and promoting mRNA degradation to reduce protein expression [32]. YTHDF3 can simultaneously promote translation and degrade m6A methylated RNA by assisting YTHDF1 and YTHDF2. Recently, enourmous studies have found that YT521-B homology domain family protein 1 (YTHDF1), as an m6A-binding protein, plays a key function in tumorigenesis of cancer [33]. Clarifying the mechanism of action of YTHDF1 is expected to find new methods for diagnosis and treatment, and can be used as one of the indicators for clinical diagnosis and prognosis evaluation [34]. This article reviews whether YTHDF1 is used as a molecular marker for clinical diagnosis or therapy, and its mechanism of action in disease occurrence.

## Diagnostic value of YTHDF1 in human cancers

**Fig. 2:**
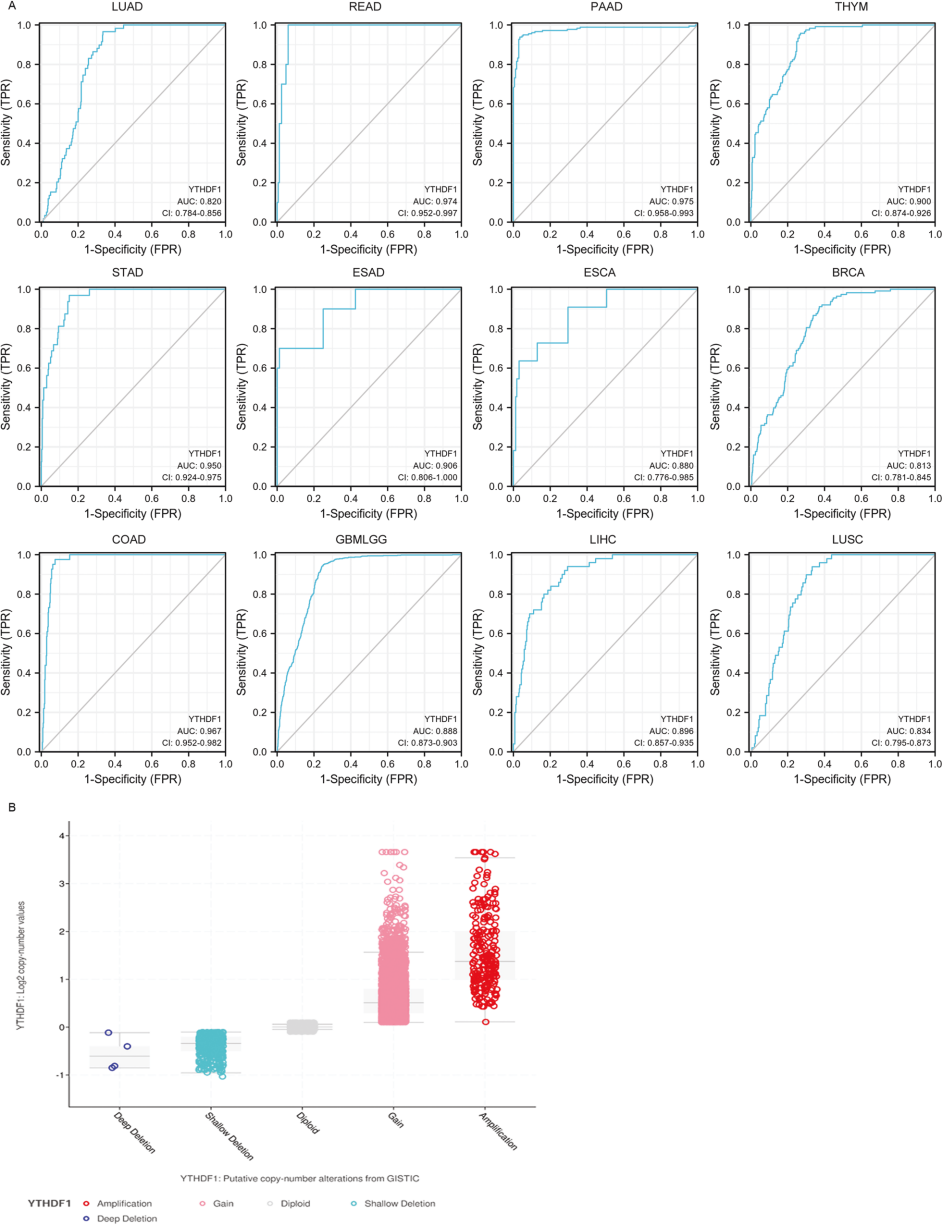
The diagnostic value of YTHDF1 in human cancers. **A** YTHDF1 was potentially used as the biomarker for early diagnosis with high specificity and sensitivity in various human cancers. LUAD, READ, PAAD, THYM, STAD, ESAD, ESCA, BRCA, COAD, GBMLGG, LIHC, and LUSC. **B** DNA copy number amplification of YTHDF1 in 10712 samples from 32 studies with various cancers based on cBioPortal database.

Although the rapid progression in cancer diagnosis in recent years, many patients with various cancers are still not effectively diagnosed at early stages. As the development of precision medicine, early and accurate diagnosis of cancers is important for effective treatment of patients with cancer. It is reported that YTHDF1 was overexpressed in different cancers, including NSCLC, breast cancers, cervical, gastric and colorectal cancers [35–39]. It has been reported that DNA copy number amplification is responsible for YTHDF1 overexpression [21]. We analyzed the amplification of YTHDF1 in 10712 samples from 32 studies with various cancers in cBioPortal database. The copy number gain and amplification (Fig. 2B & Supplement Fig. 1) were highly increased in various human cancers, which might contribute to the upregulation of YTHDF1 in cancers. Tong Liu found that YTHDF1 was obviously correlated to high-risk subtype of gastric cancer patients, and used as the biomarker for early diagnosis with high specificity and sensitivity(AUC = 0.986) [38], which suggested that YTHDF1 showed a perfect diagnostic ability in gastric cancer. To determine whether YTHDF1 was appropriate and accurate to be used as a diagnostic marker for pan-cancer, the ROC curve was performed in pan-cancer patterns from TCGA database. Diagnostic value of YTHDF1 expression in pan-cancer and corresponding paratumor tissue was evaluated by ROC curve. As shown in Fig. 2A and Table 2, the specificity and sensitivity of YTHDF1 in various tumor models were more than 0.800. All the results revealed that the YTHDF1 had a relatively high accuracy and may be possible used as a diagnostic biomarker for various human cancers.

**Table 2:** Diagnostic value of YTHDF1 in pan-cancer.

Cancer	Tumor cases	Normal cases	AUC (CI)	Cut-off value	Sensitivity	specificity
LUAD	535	59	0.820 (0.784–0.856)	6.022	0.966	0.665
READ	167	10	0.974 (0.952–0.997)	5.761	1.000	0.940
PAAD	179	171	0.975 (0.958–0.9993)	3.990	0.939	0.965
ESCA	162	11	0.880 (0.776–0.985)	6.001	0.909	0.704
THYM	119	446	0.900 (0.874–0.926)	3.780	0.958	0.731
STAD	375	32	0.950 (0.924–0.975)	5.482	0.969	0.848
ESAD	80	10	0.906 (0.806–1.000)	5.373	0.700	0.988
LIHC	374	50	0.896 (0.857–0.935)	4.982	0.940	0.706
BRCA	1109	113	0.813 (0.781–0.845)	6.239	0.912	0.628
COAD	480	41	0.967 (0.952–0.982)	5.715	0.976	0.925
GBMLGG	689	1157	0.888 (0.873–0.903)	4.607	0.949	0.749
LUSC	502	49	0.834 (0.795–0.873)	6.056	0.939	0.667

## The prognostic value of YTHDF1 in cancers

The possible prognostic value of YTHDF1 in various cancers were reviewed as follows. It has been reported that overexpressed YTHDF1 was detected, which was correlated to bad prognosis of patients with ovarian cancer [31]. It promoted tumor growth and metastasis by augmenting the translation of EIF3C in an m6A-dependent manner [31]. By analyzing the level of YTHDF1 and clinical data of breast cancer, as well as clinical specimens, it demonstrated that YTHDF1 was overexpressed in cancer cells and specimens with breast cancers. Moreover, YTHDF1 was also to be proved positively associated with tumor size, lymph node invasion and distant metastasis of cancer. Furthermore, YTHDF1 induced the progression and metastasis of breast cancer by accelerating the translation m6A-modified mRNA of FOXM1 [40]. The m6A regulators YTHDF1 and YTHDF3 were upregulated in breast cancer from TCGA and significantly associated with intrinsic subclasses and nodal metastasis, as well as poor prognosis of breast cancer patients [41]. YTHDF1 as an oncogene facilitated the progression and invasion of breast cancer cells by inducing glycolysis. YTHDF could increase tumor glycolysis by upregulating PKM2 level to finally promote tumor growth and invasion of breast cancer cells [42]. However, some researchers thought in hypoxic solid tumors, high level of YTHDF1 was associated with better clinical outcome, and knockdown of YTHDF1 induced the cancer cells resistant to cisplatin. Mechanistically, YTHDF1 deficiency suppressed cancer NSCLC progression and in vivo tumor growth via YTHDF1/Keap1-Nrf2-AKR1C1 axis [43].

Moreover, several studies on YTHDF1 were reported on liver cancers. For example, five prognostic signature for HCC was performed by Cox regression and LASSO analyses, including YTHDF1. Further experiments demonstrated that knockdown of YTHDF1 repressed the HCC in vitro and in vivo [44]. High level of YTHDF1 was closely related to poor survival of HCC. GSEA analysis showed high level of YTHDF1 might involve in cell cycle, and homologous recombination [45]. YTHDF1 was closely related to hypoxia-induced autophagy in vitro; Higher level of YTHDF1 was closely related to bad prognosis of HCC patients. HIF-1alpha-induced YTHDF1 expression was involved in autophagy-related HCC tumorigenesis through inducing translation of ATG2A and ATG14 in a m6A-dependent manner [46]. YTHDF1 was also correlated to HCC grade and it worked as an oncogene via activating the PI3K/AKT/mTOR pathway, as well as the EMT [47]. YTHDF1 promoted the progression of HCC and correlated to prognosis of HCC patients. It was also found that the expression of YTHDF1 was regulated by USF1 and c-MYC, which obviously accelerated the translation of FZD5 in an m6A-dependent manner to activate WNT/beta-catenin pathway [48]. YTHDF1 was positively associated to pathology stage. Decreased YTHDF1 contributed to better survival of HCC patients [49]. Thus, YTHDF1 might be a new therapeutic and prognostic target for cancers [49].

## The molecular mechanism of YTHDF1 in cancers

**Fig. 3:**
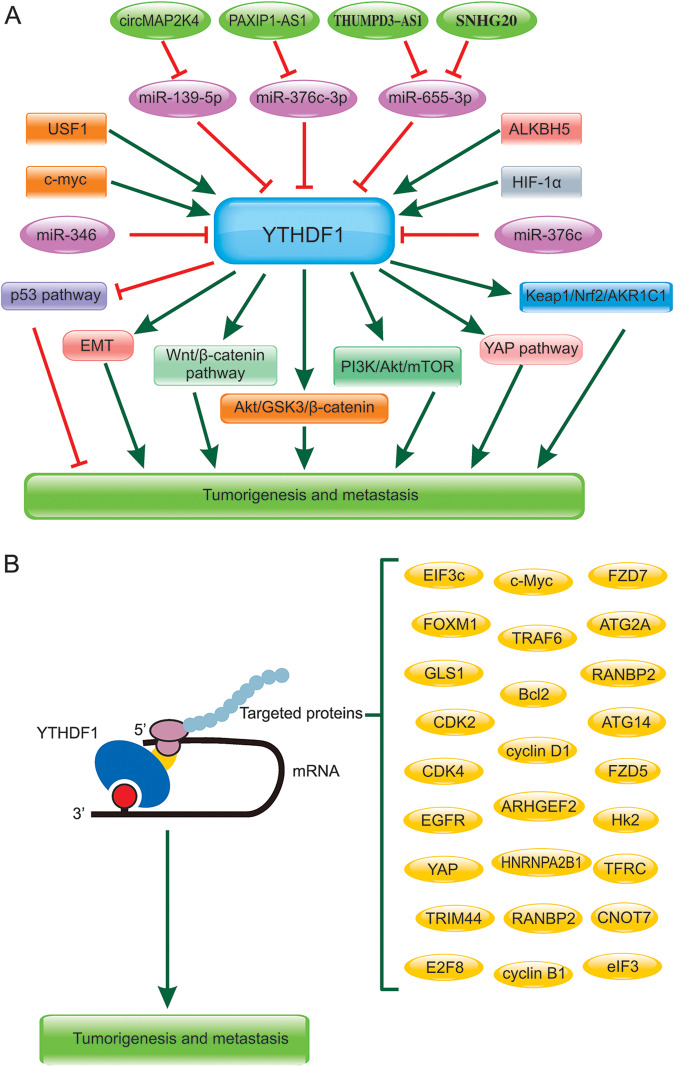
The related signaling pathway on YTHDF1 in tumorigenesis and metastasis of virous cancers. **A** The key regulatory non-coding RNA(lncRNAs, miRNAs) and proteins were shown which were involved in YTHDF1 related signaling pathways. **B** The targeted proteins regulated by YTHDF1 in tumorigenesis and metastasis.

Numerous studies demonstrated that YTHDF1 exerted an important role in tumorigenesis and metastasis of cancers by different mechanisms, such as promoting translation or regulating the stability of mRNAs [50]. Here, we reviewed the potential targeted proteins and regulatory mechanism of YTHDF1 in the tumorigenesis and metastasis of various human cancers. The related signaling pathway on YTHDF1 in cancer progression were shown in 3A and the targeted genes of YTHDF1 in various cancers were summarized in Fig. 3B and Table 3.

**Table 3:** YTHDF1 works as diagnostic biomarker and therapeutic target in patients with cancer.

Cancers	Targeted gene	YTHDF1 expression	Function
Ovarian cancer	EIF3C	Upregulated	YTHDF1 is frequently amplified and overexpressed in ovarian cancer and associated with the poor prognosis of ovarian cancer patients. Mechanismlly, YTHDF1 promoted EIF3C translation in an m6A-dependent manner [31].
Breast cancer	FOXM1	Upregulated	YTHDF1 was overexpressed in breast cancer cells and clinical specimens, which was positively correlated with intrinsic subclasses, lymph node invasion and distant metastasis in breast cancer patients [41]. It promoted FOXM1 translocation in m6A-modified mRNA [40].
Breast cancer	E2F8	Upregulated	YTHDF1 regulated E2F8 mRNA stability and DNA damage repair to promote breast tumor growth and produce chemoresistance [36].
Breast cancer	PKM2	Upregulated	YTHDF1 increased tumor glycolysis by upregulating PKM2 and promoted the tumorigenesis and metastasis potential of breast cancer cells [42].
HCC	/	Upregulated	YTHDF1 knockdown inhibited proliferation and metastasis in vitro and in vivo. YTHDF1 promoted aggressive phenotypes by inducing EMT and activating AKT/GSK3/β-catenin pathway [44]
HCC	ATG2A, ATG14	Upregulated	Overexpression of YTHDF1 in HCC tissues was associated with poor prognosis, which facilitated autophage-related HCC progression via promoting the translation of ATG2A and ATG14 [46].
HCC	FZD5	Upregulated	YTHDF1 expression was transcriptionally regulated by USF1 and c-MYC and increased the translation of FZD5 mRNA in an m6A-dependend manner in HCC [48].
CRC	GLS1	Upregulated	YTHDF1 was overexpressed in cisplatin-resistant CRC cells by interacting at the 3’ UTR of GLS1 and promoting GLS1 synthesis [65].
Gastric cancer	USP14	Upregulated	YTHDF1 was related to poor prognosis, acting as an independent prognostic factor of poor survival in GC patients. YTHDF1 promoted USP14 protein translation in an m(6)A-dependent manner [53].
Gastric cancer	FZD7	Upregulated	High expression of YTHDF1 was associated with more aggressive tumor progression and poor overall survival by promoting the translation of FZD7 in m6A-manner [66].
ICC	EGFR	Upregulated	YTHDF1 overexpression was associated with shorter survival of ICC patients. YTHDF1 increased the translation of EGFR mRNA in m6A-dependent manner and activated AKT/YAP induced tumorigenesis of ICC [54].
NSCLC	CDK2, CDK4, and cyclin D1	Upregulated	High level of YTHDF1 associated with better clinical outcome. Keap1-Nrf2-AKR1C1 axis was the downstream mediator of YTHDF1 in NSCLC [43].
Glioma	/	Upregulated	YTHDF1 played an essential function in glioma diagnosis, treatment and prognosis [61]. hsa-mir-346 negatively regulated and bind to 3’UTR of YTHDF1.
Prostate cancer	TRIM44	Upregulated	YTHDF1 promoted PCa cell proliferation, migration, and invasion by regulating TRIM44. Moreover, the prognosis of patients with PCa with high YTHDF1 was relatively poor [56].
Esophageal carcinoma	/	Upregulated	Expression of YTHDF1 was associated with multiple clinical features in ESCA patients and involved in multiple ceRNA network [59].
LUAD	Cyclin B1	Upregulated	YTHDF1 was highly expressed in KRAS/TP53-mutant patients and associated with the poor prognosis of LUAD patients by promoting translation of cyclin B1 mRNA [51].
Cervical cancer	RANBP2	Upregulated	YTHDF1 was closely associated with the poor prognosis of cervical cancer patients and regulated RANBP2 translation in an m(6)A-dependent manner [37].
Cervical cancer	HK2	Upregulated	YTHDF1 was recruited by METTL3 to enhance HK2 stability in m6A modification and promote tumorigenesis of cervical cancer [57].
CRC	ARHGEF2	Upregulated	High expression of YTHDF1 was correlated to metastatic gene signature in tumor tissues. YTHDF1 increased translation of ARHGEF2 to activate RhoA signaling and promote cell proliferation and metastasis [39].
Osteosarcoma	CNOT7	Upregulated	YTHDF1 recognized the m6A site of CNOT7 mRNA and promoted the tumorigenesis and metastasis of osteosarcoma [58].
HNSCC	TRFC	Upregulated	YTHDF1 increased the translation of TFRC mRNA in a m6A-modified manner to promote tumorigenesis depending on iron metabolism in HPSCC [52].
OSCC	c-Myc	Upregulated	METTL3 targeted the 3’ UTR of the c-Myc transcript in m(6)A- modification and accelerated the c-Myc stability to promote tumorigenesis [60].

*ICC* intrahepatic cholangiocarcinoma.

In patients with LUAD, KRAS and TP53 mutations increased the progression and metastasis of LUAD. It has been found YTHDF1 was overexpressed in KRAS/TP53-mutant LUAD patients and associated with their poor prognosis. The experimental data revealed that elevated YTHDF1 increased the cyclin B1 mRNA in an m(6)A-dependent manner to facilitate the tumor progression and adverse prognosis of KRAS/TP53-mutant LUAD patients [51]. YTHDF1 was found to regulate the iron metabolism in progression of head and neck squamous cell carcinomas (HNSCCs) in vitro and in vivo, which interacted with 3’UTR and 5’UTR of TRFC mRNA and positively regulated the TFRC mRNA translation in m6A manner [52]. Thus, how to induce the YTHDF1-mediated ferroptosis of cancer cells might be an effective way for clinical therapy of HNSCCs. YTHDF1 was proved to be high expressed in gastric cancer (GC) and worked as an independent prognosis biomarker of poor survival for GC patients. Mechanically, YTHDF1 promoted tumor progression and metastasis of GC by promoting USP14 translation [53]. YTHDF1 was overexpressed in intrahepatic cholangiocarcinoma (ICC) and closely correlated to short survival of ICC patients, which was by increasing the translation of EGFR in a m6A-dependent manner [54]. Merkel Cell Carcinoma normally outburst by the Merkel cell polyomavirus (MCPyV) and YTHDF1 has high copy gains and was highly expressed in Merkel cell carcinoma, which played an oncogenic role by promoting the translation initiation factor eIF3 [55]. YTHDF1 was highly expressed in both prostate cancer (PCa) tissues and cells, and the high level of YTHDF1 was also associated with relatively poor prognosis of PCa patients. RNA sequencing and functional experiment results showed that YTHDF1 promoted the progression of PCa by increasing the TRIM44 mRNA translation [56]. YTHDF1 was overexpressed in cervical cancer, and significantly associated with the bad prognosis of the patients. moreover, it was found that YTHDF1 regulated RANBP2 translation in an m(6)A-dependent manner [37]. YTHDF1 increased HK2 stability by m(6)A modification and promoted cervical cancer progression and Warburg effect [57]. YTHDF1 was also detected to be overexpressed in OS tissues at mRNA and protein level. YTHDF1 promoted proliferation and invasion of the OS cells. Moreover, CNOT7 could be the target of YTHDF1, which was recognized at the m6A sites of CONT7. Additionally, METTL3 also increased the m6A level of CONT7 [58].

The regulatory mechanism of YTHDF1 in human cancers were demonstrated in several cancers. For example, in ESCA patients, the level of YTHDF1 was closely related to multiple clinical indicators. It is found that YTHDF1 co-expressed genes were involved in glycolysis and ferroptosis [59]. Hsa_circ_0007456(circMAP2K4)/miR-139-5p/YTHDF1 axis was found to be a circRNA regulatory network related to YTHDF1 and promoted HCC proliferation [45]. METTL3 was overexpressed in tumor samples and correlated to the bad prognosis of oral squamous cell carcinoma patients. mechanically, YTHDF1 regulated m6A modification promoted the stability of c-Myc mRNA catalyzed by METTL3 [60]. Mir-346 negativly regulated YTHDF1, which plays a crucial role in glioma diagnosis, treatment and prognosis [61]. Most studies revealed the role of YTHDF1 regulating mRNA translation efficiency, but it was found YTHDF1 and AGO2 co-localized in P-body, and involved in miRNA-mediate mRNA degradation [62]. Higher expression of YTHDF1 were detected in NSCLC cells. miR-376c negatively modulated YTHDF1 expression and inhibited the malignant phenotypes of NSCLC cells [63]. Moreover, ALKBH5-mediated m(6)A demethylation improved the mRNA stability of YTHDF1 and increased the expression of YTHDF1 leading to the translation of YAP and contributing to cardiomyocyte proliferation and heart regeneration [64].

Furthermore, Chemoresistance remains the major obstacle for clinical therapy of cancers. YTHDF1 knockdown enhanced chemosensitivity to Adriamycin, Cisplatin and Olaparib. mechanically, YTHDF1 regulated the mRNA stability of its target molecule E2F8 and DNA damage repair in a METTL14-dependent manner to promote chemoresistance [36]. YTHDF1 was obviously increased in cisplatin-resistant colorectal cancer cells, which was interacting at 3’ UTR of GLS1, and promoting the translation of GLS1 to induce cisplatin resistance [65].

Additionally, YTHDF1 mutations normally affected the amplification in human cancers and contributed to the tumorigenesis of various human cancers. YTHDF1 was found to have mutation in nearly 7% of gastric cancer patients, and YTHDF1 overexpression was related to poor overall survival. Actually, YTHDF1 increased the translation of frizzled7 (FZD7) in an m(6)A-dependent manner, leading to hyperactivation of the Wnt/beta-catenin pathway [66]. Major mutations in YTHDF1 contributed to amplification in melanoma, by regulating important signaling pathways such as p53 [18]. DNA copy number gain of YTHDF1 is a frequent event in CRC and YTHDF1 overexpression was closely correlated to metastasis in cancer patients. ARHGEF2 is proved to be a key target of YTHDF1. YTHDF1 induced ARHGEF2 translation in colorectal cancer [39]. Single nucleotide polymorphisms in the YTHDF1 gene(rs6011668 C to T and rs6090311 A to G) also contributed to occurrence of neuroblastoma [67]. Additionally, YTHDF1 gene polymorphisms(rs6011668 C > T, rs6090311 A > G) affected Wilms tumor susceptibility [68]. The other two YTHDF1 SNPs (rs6011668 C > T and rs6090311 A > G) was to be investigated in 313 patients with HCC and 1446 controls. It was found that YTHDF1 rs6090311 A > G polymorphism reduces hepatoblastoma risk [69]. Thus, as a core factor in m6A modification, YTHDF1 possessed a global effects to target and regulate multiple genes in human cancer progression.

## YTHDF1 regulates cancer stem cells-like activity

Cancer stem-like cells normally promote tumorigenesis and metastasis of human cancers. YTHDF1 is found to regulate cancer stem cell(CSC)-like characteristic. For example, YTHDF1 regulated tumor progression and CSC-like characteristic in CRC. YTHDF1 knockdown suppressed the CRC cell’s tumorigenicity by inhibiting Wnt/beta-catenin pathway activity [21]. Another report also revealed that genetic ablation of YTHDF1 obviously blocked Wnt-related tumor gression with reduced intestinal stem cells (ISC) stemness. The studies revealed that YTHDF1 activated Wnt/beta-catenin pathway, which was dependent on the maintenance of ISCs in tumor progression [70]. YTHDF1 possessed an essential role to maintain the intestinal stem cells (ISCs), and TEAD1 was found to work as a direct target of YTHDF1. Knockdown of YTHDF1 reduced the translation of TEAD1, which also worked as a functional target of m(6)A-YTHDF1 in sustaining intestinal stemness [71].

CSCs were reported to be highly resistant to commonly used chemotherapeutic drugs and contributed to cancer recurrence and metastasis, which were associated with a poorer prognosis. TRIM29 enhanced the CSC-like characteristics of the cisplatin-resistant ovarian cancer cells. It has been found that TRIM29 was highly expressed in human cancers and correlated to cancer occurrence. Higher level of TRIM29 was correlated to the bad prognosis of the patients. TRIM29 promoted cancer progression by exhibiting the CSC-like features of cancer cells in an m6A-YTHDF1-dependent way [72].

**Fig. 4:**
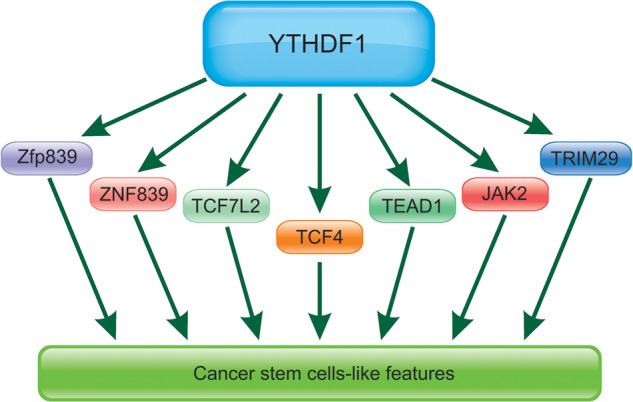
The key proteins involved in CSCs-like properties regulated by YTHDF1.

m6A has been found to regulate stem cell differentiation. Knockdown of YTHDF1 contributed to obvious impairment of cardiomyocytes (CMs) differentiation partially by inhibiting the expression of YTHDF1 [73]. Depletion of YTHDF1 led to decreased bone mass in vivo and prevented osteogenic differentiation of human bone marrow mesenchymal stem cells (hBMSCs) in vitro [74]. It has been found that ZNF839 worked as a target of YTHDF1 [74]. Additionally, JAK2 was the target of YTHDF1. METTL3 knockdown induced lower m(6)A levels of JAK2 and SOCS3 and the altered protein expressions of JAK2 and SOCS3 suppressed the activation of JAK2-STAT3 pathway [75]. Thus, we summarized the important proteins that involved in CSCs-like properties regulated by YTHDF1 in Fig. 4.

## YTHDF1 regulates immunity to promote tumor progression

It has been reported that TYHDF1 co-expressed genes mostly involved in immune response, antigen processing and presentation. YTHDF1 affected immune contexture and potentially promoted tumor progression in patients with COAD [76]. YTHDF1 is found to regulate the cross-presentation of tumor antigens in DCs and cross-priming of CD8 + T cells. YTHDF1 exerted an important role in the tumor microenvironment (TME) and YTHDF1 knockdown increased antigen-specific CD8 + T cell antitumor effects [77, 78]. Thus, YTHDF1 worked as an effective therapeutic target in immunotherapy in human cancers [77]. Moreover, in breast cancers, YTHDF1 was abnormally expressed compared with normal tissues and higher level of YTHDF1 was closely associated with poor prognosis and immune microenvironment [79]. YTHDF1 amplification is reported to be closely associated with poor overall survival in breast cancer patients. All the results demonstrated that YTHDF1 may be an effective target for clinical therapy of breast cancer [80].

However, the opposite view showed that YTHDF1 was an independent prognostic factor for recurrence-free survival. Higher level of YTHDF1 was correlated to a better prognostic outcome of NSCLC patients, much more tumor infiltrating lymphocytes (TILs), and decreased level of PD-L1 [81]. YTHDF1 was significantly overexpressed in GC compared with paired normal control tissues. Deletion of YTHDF1 contributed to recruitment of mature DCs with higher expression of MHCII and IL-12 production, which finally led to the higher production of IFN-gamma produced by infiltrated CD4(+) and CD8(+) T cells [82].

YTHDF1 were reported to regulate inflammatory response. For example, YTHDF1 recognized and bond to the m6A methylation site of SOCS3 mRNA, finally contributing to its translation and inhibiting the JAK2/STAT3 pathway, which led to the decreased production of inflammatory factors [83]. Mettl3 suppressed the apoptosis and autophagy of chondrocytes in inflammation through m(6)A/Ythdf1/Bcl2 signal pathway, for YTHDF1 regulated the stability of Bcl2 catalyzed by Mettl3 [84]. Moreover, it also promoted NLRP3 translation to regulate the inflammatory injury during endotoxic shock [85]. YTHDF1 promoted the translation of TRAF6 mRNA, which contributed to regulating intestinal immune response during bacterial infection [86].

## Conclusion and perspectives

In conclusion, YTHDF1 promoted tumorigenesis and metastasis of various human cancers normally by promoting the translation of m6A-modified mRNA. It could be used as the effective diagnostic and prognostic markers for various human cancers. Till now, several other questions were need to be explored as follows.

1. YTHDF1 was cytoplasmic and enhanced translation of targeted mRNA. It is not clearly clarified whether and how YTHDF1 regulates the programmed cell death, including ferroptosis, cell autophagy-induced cell death and pyroptosis etc.
2. T cells from YTHDF1-deficient mice produces more abundant interferon-γ, indicating that the knockout of YTHDF1 in host cells promotes T cell activation at an early stage, but the clearly molecular mechanism was not clarified till now.
3. YTHDF1 has a high clinical diagnosis accuracy and more efficient clinical diagnostic models on YTHDF1 need to be constructed and validated.

## Supplementary information

Supplement Figure Legend

Supplement Figure 1

## Data Availability

Data openly available in a public repository.
