# Supplementary material for: The potential role of m6A reader YTHDF1 as diagnostic biomarker and the signaling pathways in tumorigenesis and metastasis in pan-cancer
Source: Cell Death Discov. 2023 Jan 28;9:34. doi: 10.1038/s41420-023-01321-4 (PMC9883452; doi:10.1038/s41420-023-01321-4)
Supplement: Supplementary file 1 — Supplement Figure Legend [file 41420_2023_1321_MOESM1_ESM.docx]

Supplement Fig1. The copy number amplification, gain, diploid, shallow deletion and deep deletion were shown in various human cancers, including acute myeloid leukemia, adenosquamous, adrenocortical carcinoma, astrocytoma, breast invasive carcinoma, cervical squamous cell carcinoma, colon adenocarcinoma, dedifferentiated liposarcoma, hepatocellular carcinoma, lung cancer etc.
