# Supplementary figures and images for: The potential role of m6A reader YTHDF1 as diagnostic biomarker and the signaling pathways in tumorigenesis and metastasis in pan-cancer
Source: Cell Death Discov. 2023 Jan 28;9:34. doi: 10.1038/s41420-023-01321-4 (PMC9883452; doi:10.1038/s41420-023-01321-4)

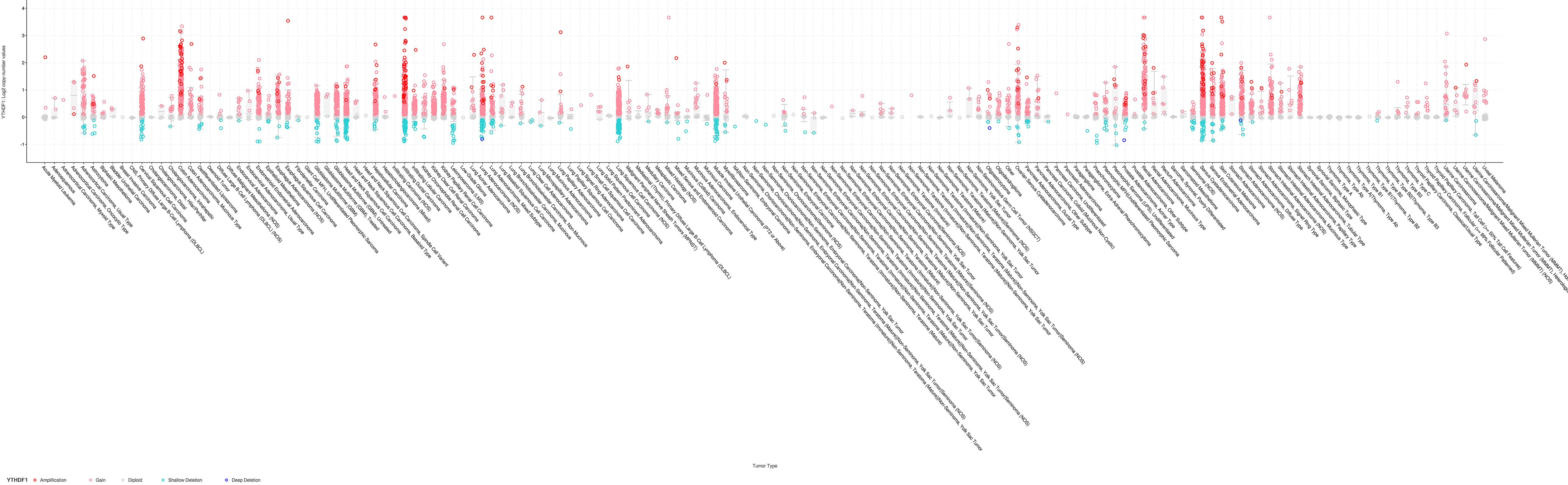

Supplement: Supplementary file 2 — Supplement Figure 1 [file 41420_2023_1321_MOESM2_ESM.tif]
